# Supplementary material for: Resolving the full spectrum of human genome variation using Linked-Reads
Source: Genome Res. 2019 Apr;29(4):635–45. doi: 10.1101/gr.234443.118 (PMC6442396; doi:10.1101/gr.234443.118)
Supplement: Supplemental Material [file supp_gr.234443.118_Supplemental_longranger-2.2.zip › longranger-2.2.tar.bz2!/lr2.2-public/lib/stan/cmdstan/stan/doc/api/html/agrad_2rev_2functions_2digamma_8hpp_source.html]

Stan: src/stan/agrad/rev/functions/digamma.hpp Source File


|  |  |
| --- | --- |
|  | Stan  2.5.0  probability, sampling & optimization |


- Main Page
- Related Pages
- Namespaces
- Classes
- Files

- File List
- File Members

All Classes Namespaces Files Functions Variables Typedefs Enumerations Enumerator Friends Macros Pages

- src
- stan
- agrad
- rev
- functions

digamma.hpp

Go to the documentation of this file.

1 #ifndef STAN\_\_AGRAD\_\_REV\_\_FUNCTIONS\_\_DIGAMMA\_HPP

2 #define STAN\_\_AGRAD\_\_REV\_\_FUNCTIONS\_\_DIGAMMA\_HPP

3

4 #include <boost/math/special\_functions/digamma.hpp>

5 #include <boost/math/special\_functions/zeta.hpp>

6 #include <stan/agrad/rev/var.hpp>

7 #include <stan/agrad/rev/internal/v\_vari.hpp>

8 #include <stan/math/functions/trigamma.hpp>

9

10 namespace stan {

11  namespace agrad {

12

13  namespace {

14  class digamma\_vari : public op\_v\_vari {

15  public:

16  digamma\_vari(vari\* avi) :

17  op\_v\_vari(boost::math::digamma(avi->val\_), avi) {

18  }

19  void chain() {

20  avi\_->adj\_ += adj\_ \* stan::math::trigamma(avi\_->val\_);

21  }

22  };

23  }

24

25  inline var digamma(const stan::agrad::var& a) {

26  return var(new digamma\_vari(a.vi\_));

27  }

28

29  }

30 }

31 #endif

stan::math::trigamma

T trigamma(T x)

**Definition:** trigamma.hpp:49

stan::agrad::var::vi\_

vari \* vi\_

Pointer to the implementation of this variable.

**Definition:** var.hpp:40

v\_vari.hpp

var.hpp

trigamma.hpp

stan::agrad::var

Independent (input) and dependent (output) variables for gradients.

**Definition:** var.hpp:27

stan::agrad::digamma

fvar< T > digamma(const fvar< T > &x)

**Definition:** digamma.hpp:16


---

[ Stan Home Page ]

*© 2011–2014,
Stan Development Team.*
